# Efficacy of acupuncture for treatment of intermittent claudication in patients with degenerative lumbar spinal stenosis: protocol for a randomized controlled trial

**DOI:** 10.1186/s13063-020-04612-8

**Published:** 2020-07-25

**Authors:** Jing Zhou, Sixing Liu, Yuanjie Sun, Weiming Wang, Zhishun Liu

**Affiliations:** 1 Department of Acupuncture, Guang’anmen Hospital, China Academy of Chinese Medical Sciences, No. 5 Beixiange, Xicheng District, Beijing, 100053 China; 2 Guiyang University of Chinese Medicine, Dongqing St., Huaxi District, Guiyang, China; 3 China Academy of Chinese Medical Sciences, Beijing, China

**Keywords:** Degenerative lumbar spinal stenosis, Neurogenic claudication, Acupuncture, Randomized controlled trial, Protocol

## Abstract

**Background:**

Degenerative lumbar spinal stenosis (DLSS) is a common condition secondary to degenerative changes. Acupuncture may be effective for treating DLSS. However, there is a lack of sufficient evidence showing the efficacy of acupuncture. The aim of this study is to assess the efficacy and safety of acupuncture for relieving neurogenic claudication in patients with DLSS.

**Methods:**

A total of 196 patients will be randomly assigned to an acupuncture group or a sham acupuncture group at a ratio of 1:1. Patients will receive 18 sessions of treatment for 6 continuous weeks. The primary outcome will be the change in the Modified Roland-Morris Disability Questionnaire score from baseline to week 6. The secondary outcomes will include the change in the scores from baseline for the Numerical Rating Scale, Swiss Spinal Stenosis Questionnaire, and Anxiety and Depression Scale. Additionally, the expectancy of acupuncture, blinding, and safety will also be assessed. All analysis will be performed based on intention-to-treat.

**Discussion:**

The aim of this study is to evaluate the efficacy and safety of acupuncture for the treatment of neurogenic claudication in patients with DLSS. A limitation of this study is that acupuncturists cannot be blinded according to the characteristics of acupuncture, which may introduce some bias.

**Trial registration:**

ClinicalTrials.gov NCT03784729 and protocol ID 2018-161-KY. Registered on 18 December 2018.

## Background

Degenerative lumbar spinal stenosis (DLSS) is a condition involving narrowing of the space for the sagittal diameter of the spinal canal or nerve root canal for the spinal nerve or cauda equina secondary to degenerative changes [1–3]. DLSS is a common cause of gluteal and lower extremity pain and more likely to affect women and elderly people aged 60–70 years [4]. Because imaging evidence is not necessarily related to clinical symptoms, there are no specific epidemiological data for DLSS [5, 6]. According to the results of magnetic resonance imaging (MRI), 30–90% of asymptomatic adults may have spinal abnormalities including disc herniation, disc degeneration, or spinal stenosis [7]. DLSS is the most common type of spinal stenosis [1, 8]. The early symptoms of DLSS are soreness and pain in the low back, gluteal region, and posterior region of the thighs, which can be relieved after resting or changing posture. As patients with DLSS experience gradually aggravated symptoms, they may have neurogenic claudication with hypoesthesia and numbness in the lateral lower legs and feet; additionally, some patients may have bowel and bladder disturbances [9, 10]. DLSS patients have a poor quality of life, especially elderly patients [11]. In accordance with the guidelines of the North American Spine Society, treatment options comprise surgical therapy, epidural steroid injections and physical therapy, and transcutaneous electrical stimulation. According to some studies, the long-term efficacy of surgery is not superior to that of non-surgical therapy [12–15]. More studies are required to explore the efficacy of non-surgical therapy [16]. According to a systematic review [17] and recent studies [18, 19], acupuncture may improve the symptoms of patients and thus their quality of life; however, there is a lack of placebo-controlled and large-sample sized studies. More studies with a sufficient sample size are needed to provide evidence of the efficacy of acupuncture for treating DLSS.

## Objectives

The aim of this study is to assess the efficacy and safety of acupuncture for relieving neurogenic claudication in patients with DLSS.

## Methods/design

### Study design

**Fig. 1:**
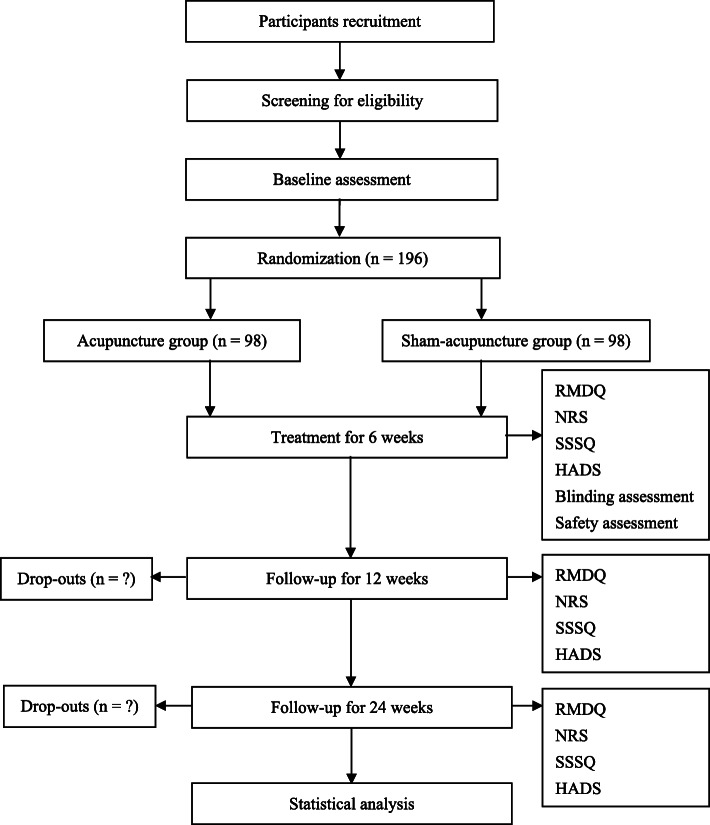
Flow chart of the study

**Fig. 2:**
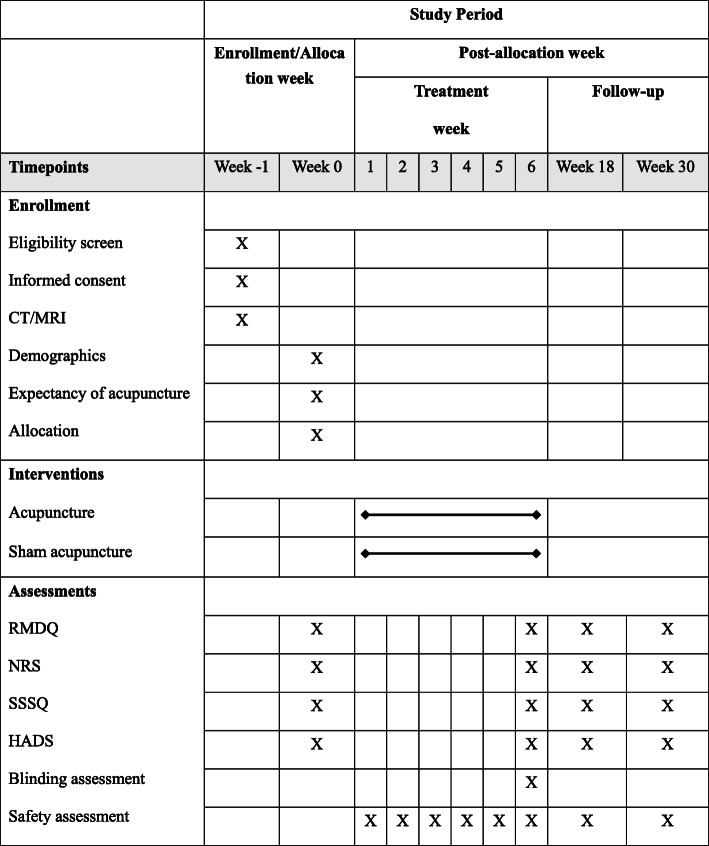
Timepoints of assessment

This study is a multicenter, randomized sham-controlled trial, and the flow chart and assessment timepoints are shown in Figs. 1 and 2. The study protocol will be drafted in accordance with the guidelines of the Standard Protocol Items: Recommendations for Interventional Trials (SPIRIT) [20]. The SPIRIT checklist is shown in the additional file. The study conforms to the principles of the Declaration of Helsinki [21] and has been approved by the Ethics Committee of Guang’anmen Hospital, China Academy of Chinese Medical Sciences (ethical number: 2018-161-KY), and other Ethics Committees of the hospitals listed below. This trial has been registered at www.clinicaltrials.gov (NCT: NCT03784729). Informed consent will be obtained from each patient before the performance of any study-specific procedure.

### Participants and recruitment

A total of 196 patients will be recruited through websites, WeChat, or posters in hospitals (Guang’anmen Hospital, Shanxi Province Hospital of Traditional Chinese Medicine, The First Hospital of Hunan University of Chinese Medicine, Guangdong Province Hospital of Traditional Chinese Medicine, and The Third Hospital of Fujian University of Chinese Medicine) from March 2019 to June 2020. The research assistants will be in charge of recruitment, and an orthopedist for each center will be in charge of the diagnosis of the patients.

### Randomization and allocation concealment

Patients who provide informed consent and are eligible will be randomly assigned at a ratio of 1:1 to either the acupuncture group or the sham acupuncture (SA) group using a central randomization system. The randomization scheme and allocation sequence will be generated by Linkermed Pharm Technology Co. Ltd. (Beijing, China). The randomization sequence will be generated in varying block sizes and stratified by center. A comprehensive document describing the randomization procedure will be kept within sealed opaque envelopes.

### Blinding

The patients, outcome assessors, and statisticians will be blinded to the treatment allocation.

## Participants

### Inclusion criteria

Participants will be eligible if they:

1. Meet the requirements for a clinical diagnosis of DLSS combined with a MRI- or computed tomography (CT)-based radiological diagnosis of central sagittal diameter stenosis of the lumbar spinal canal;
2. Have neurogenic intermittent claudication (IC) characterized by progressive pain, numbness, weakness, and tingling of the buttocks and/or legs when standing or walking or with extension of the back, which are relieved upon sitting, lying down, or bending forward [22]; they must always walk in flexion or hunchback posture;
3. Have pain of an intensity ≥ 4 in the buttocks and/or legs when walking, standing, or extending the back, as measured using the Numerical Rating Scale (NRS);
4. Have pain in the buttock and/or leg that is more severe than their pain in the lower back;
5. Have a Roland-Morris score of at least 7;
6. Have received a MRI or CT scan within 1 year that showed the anterior posterior diameter of the canal was ≤ 12 mm;
7. Are aged 50–80 years;
8. Have provided signed consent and exhibit willingness to participate in the trial.

### Exclusion criteria

Patient will be excluded if they have:

1. Congenital stenosis of the vertebral canal, indications of surgery for DLSS (e.g., segmental muscular atrophy, bowel and bladder disturbances), spinal instability requiring surgery, lumbar tuberculosis, lumbar metastatic carcinoma, or vertebral body/vertebral stenosis segment compression fracture;
2. Severe vascular, pulmonary, or coronary artery disease with limited lower extremities motility;
3. Clinical comorbidities that could interfere with the collection of data related to pain and walking function such as fibromyalgia, chronic widespread pain, amputation, stroke, Parkinson’s disease, spinal cord injury, and dementia;
4. Cognitive impairment, such that they are unable to understand the content of the assessment scales or provide accurate data;
5. A history of lumbar surgery;
6. Plans to become pregnant within 12 months or are already pregnant;
7. Received acupuncture treatments for DLSS within the previous 30 days.
8. Neurogenic IC mainly manifesting as numbness, weakness, or paresthesia of the lower extremities instead of pain.

### Patient and public involvement

The research question was first proposed by the principal investigator Zhishun Liu based on the clinical experience of treating patients with DLSS in the outpatient. Before the design of the study, some of the patients were asked if they would like to answer a series of questionnaires after treatment to evaluate their improvement. No patients were involved in the recruitment or conduct of the study. The materials of the results will be copied and given to the patients according to their request. The burden of the intervention will be assessed by patients themselves after completing the follow-up period of the study.

## Intervention

### Acupuncture group

The acupoints of bilateral Shenshu (BL23), “Dachangshu (BL25),” Weizhong (BL40), Chengshan (BL57), and Taixi (KI3) will be acupunctured. The locations of all acupoints except “BL25” will be based on the World Health Organization (WHO) Standard Acupuncture Locations [23]. “BL25” is located at horizontally outward 1.5 cun to the spinous process of the fourth lumbar vertebra. Participants will lie in the prone position and relax. A pillow will be placed under the lower abdomen of the participants, and their hands will be raised above their heads in order to maximize the enlargement of the intervertebral foramen. Prior to acupuncture, 75% alcohol pads will be used to sterilize the skin around the acupuncture points. For “BL25,” sterile disposable steel needles (0.3 mm × 75 mm) will be inserted directly to a depth of 50–70 mm until participants feel a sensation similar to electric shock radiating downward to the knees and the posterior lower legs. Then, the needle will be lifted upward for 1–2 mm without manipulation during the treatment. For the other four acupoints (BL23, BL40, BL57, and KI3), the needles (0.3 mm × 40 mm) will be inserted to a depth of 15–30 mm depending on participants’ somatotype, gently rotated and lifted three times to achieve the sense of sourness, distention, and heaviness (de qi [24]). It should be noted that the needle at KI3 will be inserted at an angle of 45° obliquely downward. The needles at all points will be retained for 30 min with light lifting, thrusting, and twirling every 10 min during each session. Participants will receive 18 treatment sessions given 3 times per week (ideally every other day) for 6 continuous weeks.

### SA group

For the SA group, needles of the same size (0.3 mm × 40 mm) will be inserted at the same acupoints used in treatment of the acupuncture group to a depth of 2–3 mm. No manipulation of needles will be conducted. The treatment duration and frequency of sessions for participants in the SA group will be the same as in the acupuncture group.

Sterile disposable steel needles (Huatuo, Suzhou, China) will be used, and acupuncturists with at least 2 years of clinical experience will apply the treatment. Patients with intolerable pain will be allowed to take 200 mg Celebrex (Pfizer China, Inc.) orally once a day for 3 consecutive days as rescue medicine with detailed record.

## Outcomes

### Primary outcome

The primary outcome will be the change in the Modified Roland-Morris Disability Questionnaire (RMDQ) score from baseline to week 6. The RMDQ [25] is a reliable pain-specific functional status questionnaire that is easy and simple for participants to complete. The RMDQ includes 24 questions with a score range of 0–24. Notably, in this study, we will modify the response to “caused by low back or leg pain” for each question, which will be more suitable for participants who have sciatica [26]. Disability is measured in terms of walking, standing, bending, working, sleeping, and activities of daily living. Higher scores indicate more severe symptoms, and a change in the score by 2.5 point is the minimal clinically important difference (MCID) for RMDQ scores [25].

### Secondary outcomes

Secondary outcomes will include the following:

1. Changes in the RMDQ score from baseline to weeks 18 and 30;
2. The proportion of participants having at least 30% and 50% reductions in the RMDQ score from baseline to weeks 6, 18, and 30;
3. Changes in the average pain scores for the buttocks and/or legs when walking, standing, or extending the back as measured by the NRS in the previous 1 week from baseline to weeks 6, 18, and 30. The NRS is a concise scale for assessing pain that is completed by the participants themselves. NRS scores range from 0 to 10 with 11 grades, and higher scores indicate greater pain [27, 28]. Two scales including one for measuring buttock and/or leg pain and another for measuring low back pain will be used (participants should answer with the average score for the previous 1 week). The degree of pain in the bilateral legs may be different, and data collection will be based on the degree of pain in the leg with more severe pain;
4. The proportion of participants having at least 30% and 50% reductions from baseline in the average pain scores for the buttocks and/or legs when walking, standing, or extending the back, as measured by the NRS for the previous 1 week at weeks 6, 18, and 30;
5. The change in the average pain score for the lower back when walking, standing, or extending the back, as measured by the NRS for the previous 1 week from baseline to weeks 6, 18, and 30;
6. Changes in the mean scores of the Swiss Spinal Stenosis Questionnaire (SSSQ) for symptom severity, physical function, and satisfaction domain from baseline to weeks 6, 18, and 30. The SSSQ is a short outcome measure for symptoms and functions. The SSSQ consists of 18 questions and three domains including symptom severity, physical function, and satisfaction with the degree of treatment. The scores for all three domains are calculated by taking the total score for the domain and dividing it by the number of answered questions, and if more than two items are missing, the scale scores for that domain are considered missing. Six questions in the symptom severity domain assess pain of the back, buttocks, legs, or feet as well as pain frequency, numbness, and weakness with scores ranging from 1 to 5, while one question assesses balance with possible scores of 1, 3, and 5. Higher scores indicate worse symptoms. The physical function domain assesses walking distance and ability to walk for pleasure, shopping, and getting around the house or apartment and from the bathroom to the bedroom. This domain has five questions with scores ranging from 1 to 4, and higher scores indicate less satisfaction. The satisfaction domain has four categories (very satisfied, somewhat satisfied, somewhat dissatisfied, and very dissatisfied) with a score range of 1–4 [29, 30];
7. The proportion of participants who are somewhat satisfied and very satisfied based on the satisfaction domain of the SSSQ at weeks 6, 18, and 30;
8. Changes in the Hospital Anxiety and Depression Scale (HADS) score from baseline to weeks 6, 18, and 30. The HADS is validated and standardized for measuring the state of anxiety and depression [31]. The HADS has two subscales with 14 items (7 items each), and a total score range of 0–21 with a range of 0–3 for each item. A score of ≥ 8 indicates the presence of anxiety and/or depression.

### Expectancy of acupuncture

Expectancy of acupuncture will be recorded at baseline. Participants will be required to answer two questions: “In general, do you believe acupuncture is effective for treating the illness?” and “Do you think acupuncture will help to improve your symptoms of DLSS?”

### Blinding assessment

Patients will be asked to answer the following questions after treatment (sessions 17 or 18) within 5 min: “Do you think you have received traditional acupuncture over the past 6 weeks?” The patients can answer “yes” or “no.”

### Safety assessment

Adverse events (AEs) related to acupuncture include severe pain, needle breakage, fainting, local hematoma, localized infection, and post-acupuncture discomfort with symptoms such as nausea, vomiting, palpitation, dizziness, headache, anorexia, and insomnia during the treatment period. AEs irrelevant to the treatment will also be recorded in detail throughout the trial.

### Data management and monitoring

Data collection and randomization will be handled by the research assistants. The double-input method will be used for data entry, and all data related to patients will be stored confidentially. All researchers and acupuncturists will take a training course before performing the study. The whole process of this trial will be conducted under the supervision of three levels of monitors. The first level of monitors composed of researchers with technical certification of acupuncture will be responsible for the whole progress of the study. The second level of monitors composed of researchers from ethics committee will be responsible for supervision of the study. The third level of monitors composed of data supervisors will ensure data authenticity. There will be no interim analyses in view of no anticipated problems that are detrimental to the participants.

## Statistical methods

### Sample size

With approximately 196 patients in the trial (98 in each group), the study is predicted to have 80% power to detect a between-group difference of 2.5 for the reduction in RMDQ score from baseline using a two-sided alpha level of 0.05 and assuming a common standard deviation of 5.52 and a dropout rate of 20%. A difference of 2.5 points on the RMDQ score was selected based on a 2–3-point change, which is the MCID recommended for sample size calculations for RMDQ scores [25].

### Statistical analysis

All data analyses will be based on the intention-to-treat principle. Missing data on the primary outcome will be imputed using the multiple imputation method under the missing at random assumption. The primary outcome, the change in the RMDQ score, will be analyzed by using analysis of covariance (ANCOVA) with baseline measures as covariates. Other continuous variables will be analyzed using ANCOVA or Wilcoxon’s rank-sum test as appropriate. We will use chi-square test or Fisher’s exact test to analyze the proportion of participants with somewhat satisfied and very satisfied in the satisfaction domain of SSSQ. AE incidences for each treatment group will be compared using chi-square or Fisher’s exact test as appropriate. The incidence rates of AEs in each treatment group will be compared using Fisher’s exact test. All statistical analyses will be performed using SAS version 9.4 (SAS Institute, Inc.) with two-sided tests at a significance level of 0.05.

### Ethics and dissemination

This study has been approved by the Ethics Committee of Guang’anmen Hospital, China Academy of Chinese Medical Sciences (ethical number: 2018-161-KY). Informed consent will be obtained from each patient before the performance of any study-specific procedure. Personal information about potential and enrolled participants will be protected and strictly confidential before, during, and after the trial. Data of the results without personal information from this study will be planned to disseminate in conferences or peer-reviewed publications.

## Discussion

Neurogenic claudication is a distinctive symptom of lumbar spinal stenosis (LSS) [32]. Meanwhile, the important consequences of DLSS are pain and poor quality of life [11]. Non-surgical management including drugs, physiotherapy, and injections is recommended for LSS before surgical intervention; however, the long-term use of painkillers remains unclear and the efficacy of physiotherapy and injections is unclear [33]. Acupuncture may be effective for improving pain and quality of life in patients with LSS [34]. It has been reported that acupuncture can influence the pain inhibitory system by causing a transient change in sciatic nerve blood flow, which circulates to the cauda equina and nerve root [35]. However, according to the results of a systematic review [17], there is no conclusive evidence of the effectiveness and safety of acupuncture for LSS, and more rigorously designed trials with a large sample size are needed. The aim of this study is to evaluate the efficacy and safety of acupuncture for the treatment of neurogenic claudication in patients with DLSS. The results of this study may provide more evidence of non-surgical therapy for LSS, and more patients may benefit from the treatment.

As for outcome assessments, the RMDQ will be selected as the primary outcome, as it is a reliable pain-specific functional status questionnaire and easy and simple for patients to complete. Additionally, a between-group difference of 2.5 points [25] of the change from baseline in the RMDQ score will be selected as MCID in this trial in order to make the results robust and reliable. Moreover, anxiety and depression were found to have a moderate inverse relationship with the severity of disability [32]. Assessing these symptoms will make the outcome measures comprehensive. Additionally, about half of DLSS patients with intermittent claudication in China may have experience of being treated with acupuncture, and it is not easy to blind the patients. Thus, minimal needling will be used in this trial instead of no penetration of the skin to avoid the absence of marks indicating penetration of the skin, which may preclude blinding of the patients. However, a limitation of this study is that acupuncturists cannot be blinded according to the characteristics of acupuncture, which may introduce some bias.

## Trial status

We are currently recruiting participants for this trial. The protocol was registered on ClinicalTrials.gov on 18 December 2018 with the identifier NCT03784729 and the unique protocol ID 2018-161-KY. The date recruitment began was March 1, 2019, and the approximate date when recruitment will be completed will be August 31, 2020.

## Supplementary information

**Additional file 1.** Standard Protocol Items: Recommendations for Interventional Trials (SPIRIT).

## Data Availability

The full data set will be made available when this trial is completed and published. Requests for the data to be released should be sent to ZSL (principal investigator). Data of the results from this study will be planned to disseminate in conferences or peer-reviewed publications.

## Abbreviations

DLSS: Degenerative lumbar spinal stenosis
MRI: Magnetic resonance imaging
SPIRIT: Standard Protocol Items: Recommendations for Interventional Trials
SA: Sham acupuncture
CT: Computed tomography
IC: Intermittent claudication
NRS: Numerical Rating Scale
RMDQ: Modified Roland-Morris Disability Questionnaire
MCID: Minimal clinically important difference
SSSQ: Swiss Spinal Stenosis Questionnaire
HADS: Hospital Anxiety and Depression Scale
AEs: Adverse events
ANCOVA: Analysis of covariance
LSS: Lumbar spinal stenosis
WHO: World Health Organization
